# *Pseudoplagiostoma* Causing Leaf Spot Disease in Key Tropical Fruit Crops in Thailand

**DOI:** 10.3390/plants13233379

**Published:** 2024-11-30

**Authors:** Sukanya Haituk, Anuruddha Karunarathna, Thitima Wongwan, Tipprapa Promthep, Sirikanlaya Sittihan, Hiran A. Ariyawansa, Chiharu Nakashima, Ratchadawan Cheewangkoon

**Affiliations:** 1 Department of Entomology and Plant Pathology, Faculty of Agriculture, Chiang Mai University, Chiang Mai 50200, Thailand; sukanya.h@cmu.ac.th (S.H.); anumandrack@yahoo.com (A.K.); thitima.wongwan@cmu.ac.th (T.W.); tipprapa_p@cmu.ac.th (T.P.); sirikanlaya_si@cmu.ac.th (S.S.); 2 Office of the Research Administration, Chiang Mai University, Chiang Mai 50200, Thailand; 3 Department of Plant Pathology and Microbiology, College of Bioresources and Agriculture, National Taiwan University, Taipei 10617, Taiwan; ariyawansa44@ntu.edu.tw; 4 Graduate School of Bioresources, Mie University, Kurima-machiya 1577, Tsu 514-8507, Mie, Japan; chiharu@bio.mie-u.ac.jp

**Keywords:** fruit crop diseases, host specificity, pathogens, plant diseases, taxonomy and phylogeny

## Abstract

Fruit crops have a pivotal role in Thailand’s economy. Continuous evaluation of 13 potential and emerging diseases in fruit crops is important. Necrotic and discolored leaf spots were observed in *Persea americana* (avocado) and *Mangifera indica* (mango). The isolated fungi from the leaf spot were identified through multi-loci phylogenetic analyses using a concatenated matrix of ITS, LSU, *tef1α*, and *tub2*. The fungal isolates from *Pe. americana* were identified as *Pseudoplagiostoma perseae*, and isolates from *Mangifera indica* were identified as *Ps. mangiferae*. The pathogenicity assays confirmed that *Ps. perseae* causes leaf spots of *Pe. americana*, while *Ps. mangiferae* causes leaf blotch of *M. indica.* The pathogenicity of *Ps. perseae* and *Ps. mangiferae* has been reported in China and Taiwan. Hence, this study provides a report of the novel geographical distribution of *Ps. perseae* and *Ps. mangiferae*. Moreover, the cross-inoculation tests of *Ps. perseae* and *Ps. mangiferae* on *M. indica* and *Pe. americana* were conducted, respectively. Both pathogens showed host specificity, as suggested by the phylogenetic relationship and the host plants. In addition, disease control with carbendazim, trifloxystrobin, mancozeb, and prochloraz was assayed. All fungicides equally inhibited the mycelial growth of both pathogens.

## 1. Introduction

The fruit industry provides a significant contribution to the Thai economy due to the high fruit crop diversity in the hostile biogeography of Thailand [1,2]. Evergreen *Persea americana* (Avocado) belongs to *Lauraceae* and is a high-demand commercial crop worldwide due to its nutrient profile and versatile applications in gastronomy. *Persea americana* cultivation in Thailand was initiated in the early 1900s in Nan province and extended to the north and northeast areas, namely Chiang Rai, Chiang Mai, Nakhonratchasima, and Chaiyaphum [3,4]. These days, the Asia–Pacific region has the biggest market for *Pe. americana* produced in Thailand [5]. On the other hand, *Mangifera indica* cultivation in Thailand dates back to the early historic era [6]. Currently, 174 cultivars of *M. indica* are known from Thailand and cropping through Thailand according to the aptitude of environmental conditions [6].

Recent studies on fruit crop pathogens from Thailand have revealed several emerging fungal pathogens such as *Parvodontia austrosinensis* and *Paramyrothecium* spp., which changed the niches, from saprophytes to pathogens [7,8]. Hence, continuous assessment and proper investigations of diseases are necessary for the detection and management of diseases. During the field survey of fruit tree diseases in northern and northeastern Thailand, plant pathogenic species of the fungal genus *Pseudoplagiostoma* on *Persea americana* and *Mangifera indica* were observed.

The genus *Pseudoplagiostoma*, *Pseudoplagiostomataceae*, *Diporthales*, was established as a coelomycetous fungus on eucalypts [9,10] and currently comprises seventeen species including *P. alsophilae*, *P. bambusae*, *P. castaneae*, *P. corymbiae*, *P. corymbiicola*, *P. dipterocarpi*, *P. dipterocapicola*, *P. eucalypti*, *P. inthanonense*, *P. jasmine*, *P. machili*, *P. mangiferae*, *P. myracrodruonis*, *P. oldii*, *P. perseae*, *P. variabile*, and *P. wuyishanense* [11]. The sexual morph *Pseudoplagiostoma* is morphologically characterized by horizontal, dark, soft-textured perithecial ascomata lacking stromatic tissues but with a lateral ostiolar neck; distinct non-amyloid asci with a refractive apical ring; eight 1-septate ascospores, which have elongated appendages at both ends, but lacking true paraphyses. The asexual morph is characterized by superficial and immersed pycnidial or acervular conidiomata; aseptate ellipsoid, hyaline, smooth conidia, sporulating from phialidic or annellidic conidiogenous cells emerging from poorly developed stromata [9,10].

*Pseudoplagiostoma* species are mainly recognized as a plant pathogen causing leaf spots and early defoliation of arboreal and shrub plants, *Castanea mollissima*, *Eucalyptus* spp., *Jasminum grandiflorum*, *Mangifera indica*, *Persea americana*, while some species are categorized as saprobes and endophytes. Furthermore, they are distributed in Australia, Bhutan, Brazil, China, Malaysia, Taiwan, Thailand, the USA, Venezuela, and Vietnam [10,12,13,14,15,16].

Due to the economic importance of *Mangifera indica* and *Persea americana*, evaluation of successful disease control is important. Chemical fungicide is one of the most convenient methods for disease management. However, excessive chemical fungicide usage causes health and environmental repercussions [17]. Hence, the minimum effective dose for carbendazim, trifloxystrobin, mancozeb, and prochloraz was investigated for sustainable disease control.

Under the current survey of fruit crop diseases in Thailand, leaf spots were observed on *Mangifera indica* and *Persea americana*. To identify the causal pathogen, the potential fungal pathogens were isolated using single spore isolation and identified using a polyphasic approach. The Koch’s postulates-based pathogenicity assay identified the pathogens as *Pseudoplagiostoma perseae*, and *Ps. Mangiferae.* The pesticide sensitivity of both pathogens towards Mancozeb, Carbendazim, Prochloraz, and Trifloxystrobin was studied, and all pesticides could control the pathogens in the recommended concentration.

## 2. Results

### 2.1. Field Survey and Sample Collection

#### 2.1.1. Foliar Disease on *Perse Americana*

Leaf samples with leaf spots were collected from orchards located in Hang Dong district (18.79620° N, 98.81091° E), Mae Tang district (19.19018° N, 98.86153° E), and Mae On district (18.85546° N, 99.27952° E), Chiang Mai Province, Thailand, on January 2024. The symptoms initiate with small brown spots with a yellow halo on the upper leaf surface. The lesions are expanded, and the center of the lesion becomes dark brown. Later, it is coalescent and irregular in shape. Conidiomata are visible as small brackish dots on the lesion. Four isolates were obtained from the symptomatic leaves.

#### 2.1.2. Foliar Disease on *Mangifera Indica*

Leaf samples with leaf spots were collected from the Agriculture Innovation Research, Integration, Demonstration, and Training Center (18.76324° N, 98.93190° E), Chiang Mai University, Chiang Mai, on January 2024. The small brown spots with a yellow halo on the upper leaf surface were observed as a primary symptom. The lesions were expanded, and the margin of the lesions became dark brown. Later, they became coalescent and irregular in shape. Conidiomata were visible as convex domes in the brown spots. Two isolates were obtained from the symptomatic leaves.

### 2.2. Identification of the Fungi Associated with Symptoms

#### 2.2.1. Phylogenetic Analysis

**Figure 1:**
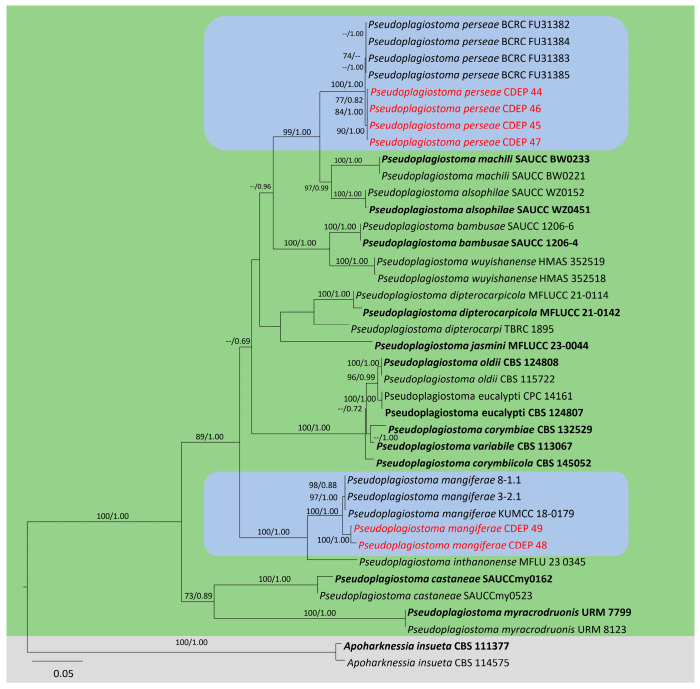
A maximum likelihood (ML) tree of the *Pseudoplagiostoma* species based on a concatenated matrix of LSU, ITS, *tef1α*, and *tub2* gene sequences with *Apoharknessia insueta* as outgroup. Bootstrap support values for ML analyses higher than 60%, and Bayesian posterior probabilities (BYPP) greater than 0.80 are indicated as above the nodes, respectively. Ex-type cultures are indicated in bold. Strains obtained in this study are in red.

The concatenated matrix of LSU, ITS, *tef1α*, and *tub2* gene nucleotide sequences comprised 2445 characters (LSU: 831, ITS: 678, *tef1a*: 393, *tub2*: 543). Maximum likelihood (ML) phylogenetic analysis of concatenated data set generated the best scoring tree final ML optimization likelihood value of −13,823.171886. The estimated base frequencies for the GTR+I+G model of the combined data set were A—0.230968, C—0.268816, G—0.266651, and T—0.233565. The substitution rates were AC—1.631648, AG—2.121990, AT—1.369644, CG—0.708257, CT—4.725609, and GT—1.000000. Furthermore, the proportion of invariable sites I—0.525980 and gamma distribution shape parameter 1.070784. The matrix had 1129 distinct alignment patterns, with 22.52% of undetermined characters or gaps. The best-scoring ML tree is given in Figure 1.

The ML and Bayesian posterior probability phylogeny shows that the isolates CDEP-44, CDEP-45, CDEP-46, CDEP-47, CDEP-48, and CDEP-49 were located in *Pseudoplagiostoma*. The isolates CDEP-44, CDEP-45, CDEP-46, and CDEP-47, form a stable sister clade with *Ps. perseae*, with 100% ML and 1.0 BYPP support (Figure 1) while CDEP-48 and CDEP-49 form a stable sister clade with *Ps. mangiferae*. Hence, phylogenetically the isolates CDEP-44, CDEP-45, CDEP-46, and CDEP-47, represent *Ps. perseae* while, CDEP-48 and CDEP-49 represent *Ps. mangiferae.*

#### 2.2.2. Taxonomy

**Figure 2:**
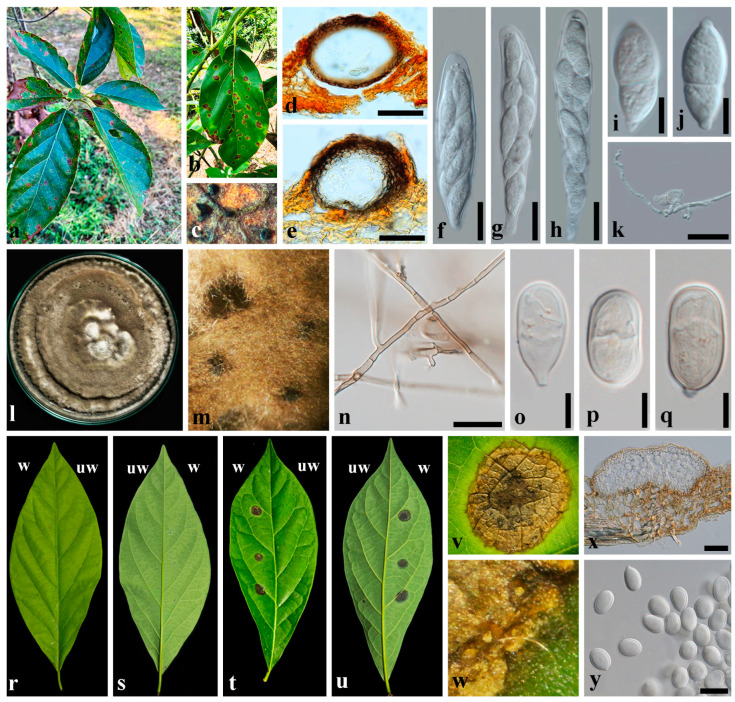
*Pseudoplagiostoma perseae* (**a**–**q**); (**a**,**b**) leaf spot symptoms of *Persea americana*; (**c**) conidiomata on a lesion; (**d**,**e**) cross-section through ascomata; (**f**–**h**) asci; (**i**,**j**) ascospores; (**k**) germination of ascospore; (**l**) mycelial colony on PDA; (**m**) ascomata on PDA; (**n**) colored hyphae on PDA; (**o**–**q**) conidia with an indistinct septum; (**r**–**u**) pathogenicity test for *Ps. perseae* CDEP-44 against *Pe. Americana*, w, wounded; uw, unwounded; (**r**) the upper surface of a leaf inoculated with sterile water (control); (**s**) the lower surface of a leaf inoculated with sterile water (control); (**t**) reproduced symptoms on the upper surface of a leaf inoculated with isolate CDEP-44; (**u**) reproduced symptoms on the lower surface of a leaf inoculated with isolate CDEP-44; (**v**) magnified symptoms reproduced by inoculation test; (**w**) induced conidiomata with conidial masses droplets in yellow, 3 weeks after inoculation; (**x**) vertical section of conidioma filled with conidia developed on a lesion. Scale bars: (**d**,**e**,**k**,**n**) = 20 µm; (**f**–**h**,**x**,**y**) = 10 µm; (**i**,**j**,**o**–**q**) = 5 µm.

***Pseudoplagiostoma perseae*** C.J. Wu, J.L. Chen, S.S. Tzean & H.F. Ni, *European Journal of Plant Pathology* 6: 622 (2024). Figure 2.

Index Fungorum number: 839192

Symptoms amphigenous, circular to irregular, dark brown with a yellow halo, scattered, sometimes enlarged and coalescent, 2–15 mm. Conidiomata were visible as small brackish dots on the lesion (Figure 2a–c). **Sexual morph** on *Persea americana*: Ascomata perithecial, depressed globose to subglobose, brown to black, immersed, epidermal, solitary, 86–99 × 73–86 μm ($\overset{-}{x}$ = 92 × 79 µm, *n* = 10) (Figure 2d,e); ascomatal wall consisting of three to four layers of brown to dark brown *textura angularis*; ostioles lateral, neck internally lateral lined; paraphyses absent (Figure 2d,e). Asci unitunicate, clavate to fusiform, rounded at the apex, sessile, forming apical rings, 8-spored, hyaline, 35–48 × 6–8.5 μm ($\overset{-}{x}$ = 42 × 7 µm, *n* = 20) (Figure 2f–h). Ascospores fusiform, 1-septate, two-celled unequally, upper cell longer and wider, smooth or rough, constricted at the septum, papillate (short-rostrate) at both ends, hyaline, 8.5–11 × 3–4.2 μm ($\overset{-}{x}$ = 10 × 4 µm, *n* = 40) (Figure 2i,j). **Asexual morph** produced on PDA culture: conidiomata pycnidial, subepidermal or growing on the surface, depressed globose to subglobose, solitary, brown to black, with pale yellow to cream droplet containing conidial masses, 124–203 × 59–72 μm ($\overset{-}{x}$ = 163 × 66 µm, *n* = 10) (Figure 2l,m); conidiomatal wall thin, consisting of one to two layers of *textura angularis* (Figure 2x). Conidiophore indistinct, reduced to conidiogenous cells. Conidiogenous cells cylindrical to ampuliform, collateralis densely, hyaline, smooth, proliferating percurrently, annelidic, discrete, 8.5–13.5 × 3.3–4.5 μm. Conidia sporulating blastically, solitary, acrogenous, rhexolytic, aseptate or septate, globose to irregularly globose, ellipsoid, hyaline, rounded at the apex, 10–20 × 7–13 μm ($\overset{-}{x}$ = 15 × 10 µm, *n* = 40) (Figure 2n); hulum observed at the basal end of conidia, hyaline. **Culture characteristics**: colony on potato dextrose agar (PDA) for 10 days, the colony of every isolate was circular, grew up from the surface, brown to dark brown, and induced conidiomata after culture one month.

**Figure 3:**
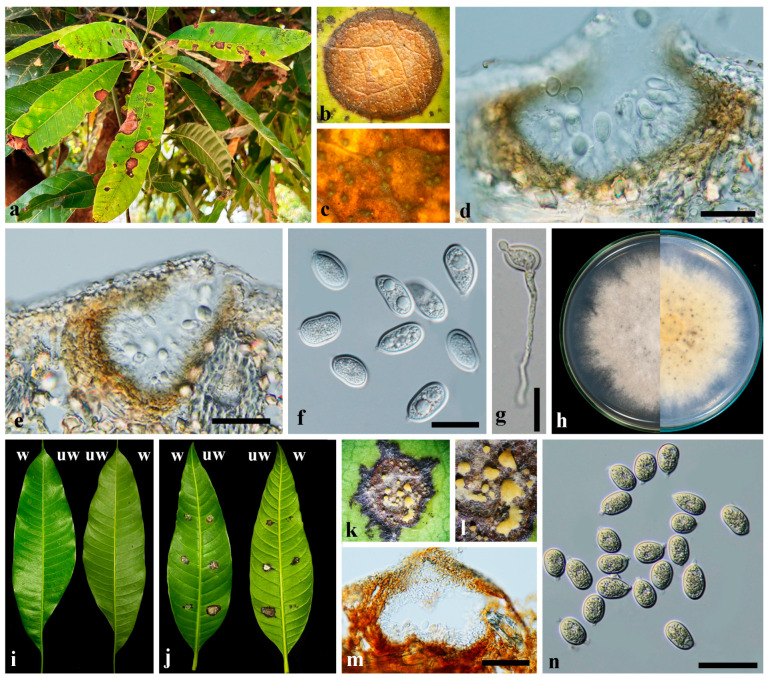
*Pseudoplagiostoma mangiferae* (**a**–**n**); (**a**) leaf spots symptoms of *Mangifera indica*; (**b**) magnified symptom; (**c**) conidiomata formed densely on a lesion; (**d**,**e**) cross-section through conidiomata; (**f**) conidia; (**g**) germination of ascospore; (**h**) colony on PDA; (**i**–**n**) pathogenicity test of *Ps. mangiferae* on *M. indica*; w, wounded; uw, un-wounded; (**i**) the upper and lower surface of a leaf inoculated with sterile water (control); (**j**) reproduced symptoms on a leaf inoculated with isolate CDEP-48; (**k**,**l**) droplets of conidial masses exudated from conidiomata on inoculated leaf, three weeks after inoculation; (**m**) conidioma formed on a inoculated leaf; (**n**) conidia from a conidial masses. Scale bars: 20 µm.

***Pseudoplagiostoma mangiferae*** Dayar., Phookamsak & K.D. Hyde, *Fungal Diversity*. 95: 121 (2019). Figure 3.

Index Fungorum number: IF555434

Symptoms amphigenous, circular to irregular with easily torn centers, are cream to light brown in color with a dark brown margin on both leaf surfaces. Fungal bodies are visible as blackish convex domes on lesions (Figure 3a–c). **Sexual morph** was not observed in this study. **Asexual morph** conidiomata pycnidial, solitary to aggregated, epidemal, submerged, erumpent, globose to depressed globose, or irregular, with yellow to cream droplet containing conidial masses, olivaceous brown to black, 102–125 × 72–140 µm ($\overset{-}{x}$ = 105 × 125 µm, *n* = 10) (Figure 3d,e); conidiomatal wall consisting three to five layers of *textura angularis*, brown to pale olivaceous brown. Conidiophores reduced to conidiogenous cells. Conidiogenous cells ampliform, cylindrical, determinate, enteroblastic, appearing as phialides with periclinal thickening or shortly proliferating percurrently at the apical part with collarette, discrete, collateralis, arising from the inner layer of conidiomatal wall, hyaline, smooth, cylindrical to ampulliform, and wider at the base, 3–10 × 4–9 µm ($\overset{-}{x}$ = 7 × 5 µm, *n* = 30) (Figure 3e). Conidia sporulating blastically, solitary, acrogenous, rhexolytic, aseptate, ellipsoidal, guttulate, smooth, thick-walled, rounded at the apex, hyaline, 20–25 × 9–13 µm ($\overset{-}{x}$ = 23 × 12 µm, *n* = 50); hilum at the base protruding (Figure 3f). **Culture characteristics**: Colonies on PDA reaching 60–80 mm diam after four weeks at room temperature, pale yellowish to cream with moderate aerial mycelia and undulated margin; reverse similar; surface slightly rough with tufts of hyphae, edge entire, wooly to cottony (Figure 3h).

### 2.3. Pathogenicity Test

#### 2.3.1. Pathogenicity of *Pseudoplagiostoma perseae* to *Persea americana*

The isolates of *Pseudoplagiostoma perseae*, CDEP-44, CDEP-45, CDEP-46, and CDEP-47, were isolated from the disease symptoms of *Persea americana* (Figure 2l,m). As a result of the inoculation test, first, the brown leaf spots appeared seven days after the inoculation (DAI). Later, the center of the lesion turned dark brown with a yellow halo. The pycnidia in brown to blackish were observed on the lesions 21 DAI. From the pycnidia, pale yellow to cream droplets containing conidial masses were exuded.

#### 2.3.2. Pathogenicity of *Pseudoplagiostoma mangiferae* to *Mangifera indica*

The isolates of *Pseudoplagiostoma mangiferae*, CDEP-48, and CDEP-49, were obtained from the lesion of *Mangifera indica* (Figure 3a). The inoculated plants with isolate CDEP-48 showed the symptoms same as the original symptomatic samples 7 DAI (Figure 3i,j). The pycnidia in blackish were observed on the lesions 21 DAI. From the pycnidia, yellow to cream droplets containing conidial masses were exuded.

#### 2.3.3. Cross Inoculation for the Host Specificity

*Pseudoplagiostoma mangiferae* isolates CDEP-48 and CDEP-49 inoculated onto *Persea americana*, and *Pseudoplagiostoma perseae* isolates CDEP-44, CDEP-45, CDEP-46, and CDEP-47 onto *Mangifera indica* did not produce any symptoms even 14 DAI .

### 2.4. Chemical Control of Pseudoplagiostoma mangiferae and Pseudoplagiostoma perseae

Four fungicides extensively used in Thailand (mancozeb, carbendazim, prochloraz, and trifloxystrobin) exhibited high efficacy in controlling the mycelial growth of *Pseudoplagiostoma mangiferae* and *P. perseanum* at the recommended dosage. All four demonstrated 100% inhibition of mycelial growth compared with the control, highlighting their robust potential in managing these fungal pathogens effectively. The findings showed that these fungicides are applicable to avocados and mangoes in Thailand to prevent leaf spots caused by *Pseudoplagiostoma* species.

## 3. Discussion

Currently, the genus *Pseudoplagiostoma* consists of seventeen species. Interestingly, *Pseudoplagiostoma* species are mainly found in arborescent plants (Table 1) and belong to *Anacardiaceae*, *Dipterocarpaceae*, *Fagaceae*, and *Myrtaceae*. The geographical habitats of these fungi are mostly tropics and subtropical regions. This study reports novel geographic habitats for *P. perseae* and *P. mangiferae.* Moreover, the ecological niches are multiple. Most species are recognized as plant pathogens, followed by endophytes and saprobes. Furthermore, from the results of phylogenetic analyses, it is suggested that each *Pseudoplagiostoma* species has host specificity, which is located in a well-supported clade composed of a specific host plant genus. Hence, *Pseudoplagiostoma* may provide a good source for studying the life mode shift among the pathogens, endophytes, and saprobes, following the speciation. The plant genus *Eucalyptus* and *Myrtaceae*, can be thought of as a harbor for multiple species of *Pseudoplagiostoma* pathogens in the course of host expansion or mode shift, and several species are reported from disjunct continents (Table 1, Figure 1).

Under the current study, the effective control measures for *Pseudoplagiostoma perseae* and *Ps. mangiferae* through chemical pesticides were investigated. For the control of these diseases, systemic fungicides: carbendazim, trifloxystrobin, and prochloraz, and contact fungicide mancozeb were suitable for practical application.

The leaf spots caused by *Pseudoplagiostoma mangiferae* and *Ps perseae* are similar to leaf spots caused by *Colletotrichum* species [16]; which easily misleads the farmers in diagnosing *Pseudoplagiostoma* leaf spot disease. This causes most farmers to tend to control the *Pseudoplagiostoma* leaf spots through chemical control prescribed for anthracnose diseases. Hence, it is important to differentiate the leaf spots caused by *Pseudoplagiostoma* species having a host specificity and anthracnose leaf spots caused by *Colletotrichum* species having a wide host range for the effective control of the diseases. These accurate diagnoses and evidence-based control of diseases lead to a reduction in excessive chemical usage and an increase in farmers’ income.

## 4. Materials and Methods

### 4.1. Sample Collection and Fungal Isolation

Symptomatic leaves of *Persea americana* were collected from the avocado orchard in Mae On District, Mae Tang District, and Jom Thong District, Chiang Mai Province, Thailand, in January 2024. That *Mangifera indica* was collected from Agriculture Innovation Research, Integration, Demonstration and Training Center, Chiang Mai University, Chiang Mai. Specimens were taken to the laboratory in plastic bags and observed under a Stemi 305 Zeiss stereo microscope. The leaves that show symptoms are taken to isolate the pathogen using the spore-shooting technique outlined by Haituk et al. [25] and used in pure cultures. Herbarium specimens were deposited in the collection of the Department of Entomology and Plant Pathology (CDEP), Faculty of Agriculture, Chiang Mai University, Chiang Mai, Thailand.

### 4.2. Fungal Identification

#### 4.2.1. Morphological Studies

The isolates were cultured on potato dextrose agar (PDA) for one month at 25 °C. Induced ascomata on the culture were studied by the hand sections. Microscopic characters were observed with the hand sections mounted in lactic acid and photographed under Axiovision Zeiss Scope-A1 microscope (Zeiss, Jena, Germany). Measurements were made with the Tarosoft (R) Image Frame Work program (Tarosoft, Bangkok, Thailand).

#### 4.2.2. DNA Extraction, PCR Amplification and Sequencing

Fungal mycelia were grown on PDA at 25–30 °C for 14 days. Genomic DNA was extracted using the DNA Extraction Mini Kit (FAVORGEN, Ping-Tung, Taiwan) following the manufacturer’s instructions. DNA sequences of five loci, including the internal transcribed spacer regions with the intervening 5.8S nrRNA gene (ITS), the partial large subunit nrRNA gene (LSU), the partial translation elongation factor 1-alpha gene (*tef1a*), and the partial beta-tubulin gene (*tub2*) were amplified by the primer pairs and polymerase chain reaction (PCR) programs listed in Table 2. Amplification reactions were performed in a 25 μL reaction volume, which contained 12.5 μL of master mix (Quick Taq HS DyeMix, TOYOBO, Osaka, Japan) 1 µL of each forward and reverse primer (Macrogen Inc., Seoul, Republic of Korea), 9.5 µL of PCR-grade water, and 1 µL of DNA template. PCR amplification products were visualized on 1% agarose electrophoresis gel. The PCR products were sequenced using Sanger sequencing by First Base Company (Kembangan, Malaysia). The obtained nucleotide sequences were deposited in GenBank (accession numbers available in Table 3).

#### 4.2.3. Phylogenetic Analysis

The DNA sequences obtained in this study were assembled and aligned with 39 sequences of *Pseudoplagiostoma* species retrieved from previous studies by Zhang et al. [13] and were aligned with MAFFT v.7.036 (http://mafft.cbrc.jp/alignment/server/), accessed on 13 May 2024). [30] and edited manually using MEGA 7 software package [31]. Data were converted from fasta to nexus and PHYLIP files format using Alignment Transformation Environment online, https://sing.ei.uvigo.es/ALTER/, accessed on 13 May 2024) [32]. Multilocus phylogenetic analyses were based on the algorithm maximum likelihood (ML) and Bayesian inference (BI) methods. The ML was run on the CIPRES Science Gateway portal (https://www.phylo.org, accessed on 12 March 2024) [33] using RaxML–HPC2 on XSEDE v. 8.2.12 [34,35] and employed a GTR+I+G model of nucleotide substitution, with 1000 bootstrap replications. The best-scoring tree from ML analysis was used to represent the phylogenetic tree and the resulting tree was visualized in FigTree v.1.4.3 [36].

Bayesian inference (BI) analysis to evaluate posterior probabilities (BYPP) [37,38] through Markov Chain Monte Carlo sampling (MCMC) was performed in MrBayes on XSEDE, MrBayes 3.2.6 [39] using the CIPRES Science Gateway platform [34]. Six simultaneous Markov chains were run for 1,000,000 generations with two parallel runs and trees were sampled every 100th generation. The first 25% of generated trees representing the burn-in phase were discarded and the remaining trees were used to calculate posterior probabilities of the majority rule consensus tree. The results were combined and visualized in best scoring ML tree.

### 4.3. Pathogenicity Test and Cross Inoculation for the Host Specificity

Koch’s postulates are used to confirm the pathogenicity of pathogens in their original hosts. Under the current study to prove the pathogenicity through Koch’s postulates, isolates CDEP-44, CDEP-45, CDEP-46, and CDEP-47 were inoculated on their original host *Persea americana*. Further, isolates CDEP-48, and CDEP-49 were inoculated on their original host *Mangifera indica.* Nevertheless, in the current study, the ability of CDEP-44, CDEP-45, CDEP-46, and CDEP-47 to infect *Mangifera indica* was tested. Furthermore, the ability of CDEP-48 and CDEP-49 to infect *Persea americana* was also tested. For both sets of experiments, the following methodology was used: The experiments were carried out under controlled laboratory conditions on detached leaves, following the methodology outlined by Withee et al. [8]. Healthy leaves were surface disinfected with 70% ethanol, washed twice with sterile distilled water, and air-dried under laminar flow. Conidial suspensions (10^6^ conidia/mL) were prepared for all fungal isolates in sterile distilled water. The conidia (10 μL of spore suspension) were placed on the upper surface of the leaves. In addition, the leaves were also wounded before inoculation. The upper epidermis was wounded approximately 2 cm from the mid-vein by pricking with a sterile needle to about 1 mm depth. Five wounds were made for each leaf, vertically on each side of the mid-vein. Control leaves received drops of sterile distilled water. All inoculated leaves were placed in a moist chamber at 28–30 °C under daylight conditions. After 3 weeks, symptoms were recorded, compared, and confirmed with the original morphology and molecular relationships.

### 4.4. Fungicide Sensitivity Testing

Mancozeb (60% WG, Sotus International Co., Ltd.) (Nonthaburi, Thailand), Carbendazim (50% WP; Sa-haphon Kemikaset Ltd.) (Bangkok, Thailand), Prochloraz (50% WP; Bayer Holding (Thailand) Co., Ltd.) (Bangkok, Thailand) and Trifloxystrobin (25% WP; Bayer Holding (Thailand) Co., Ltd.) (Bangkok, Thailand). were dissolved in distilled water to obtained stock a solution of 250, 100, 100 and 25 mg/mL, respectively. Mancozeb has a multi-site activity (M03), carbendazim is a methyl benzimidazole carbamates (MBC, FRAC1), prochloraz is a demethylation inhibitor (DMI, FRAC3), and trifloxystrobin is a quinoneoutside inhibitor (QoI, FRAC11). All solutions were stored at 4 °C in the dark until needed. The sensitivity of the *P. mangiferae* (CDEP-48) and *P. perseae* (CDEP-44) to four fungicides were determined (in vitro) by transferring mycelium plugs to PDA plates containing mancozeb (1500 µg/mL), carbendazim (500 µg/mL), prochloraz (500 µg/mL), and trifloxystrobin (250 µg/mL). PDA plates without any fungicide were considered as the control. Five replicates for each fungicide were performed. The cultures were incubated at 25 °C for 14 days. The diameters of the colonies were calculated for the percentage inhibition of mycelial growth (PIMG) from the following formula [(mean diameter on control (mm) − mean diameter on PDA amended fungicide (mm))/mean diameter on control (mm)] × 100.

## Figures and Tables

**Table 1:** Host, habitat and distribution of *Pseudoplagiostoma species*.

Species	Host	Family	Life Mode	Reference
*Pseudoplagiostoma alsophilae*	*Alsophila spinulosa*	Cyatheaceae	pathogenic	[13]
*P. bambusae*	*Bambusoideae* sp.	Poaceae	pathogenic	[13]
*P. castaneae*	*Castanea mollissima*	Fagaceae	Saprobic	[12]
*P. corymbiae*	*Corymbia* sp.	Myrtaceae	pathogenic	[18]
*P. corymbiicola*	*Corymbia citriodora*	Myrtaceae	pathogenic	[19]
*P. dipterocarpi*	*Dipterocarpus tuberculatus*	Dipterocarpaceae	endophytic	[14]
*P. dipterocarpicola*	*Dipterocarpus* sp.	Dipterocarpaceae	Saprobic	[20]
*P. eucalypti*	*Eucalyptus camaldulensis*	Myrtaceae	pathogenic	[10]
	*E. urophylla*	Myrtaceae	pathogenic	[10]
*P. mangiferae*	*Mangifera* sp.	Anacardiaceae	pathogenic	[16,21]
*P. myracrodruonis*	*Astronium urundeuva*	Anacardiaceae	endophytic	[15]
*P. machili*	*Machilus nanmu*	Lauraceae	pathogenic	[13]
*P. oldii*	*Eucalyptus camaldulensis*	Myrtaceae	pathogenic	[10]
*P. Perseae*	*Persea americana*	Lauraceae	pathogenic	[22]
*P. variabile*	*Eucalyptus globulus*	Myrtaceae	pathogenic	[10]
*P. jasmini*	*Jasminum grandiflorum*	Oleaceae	pathogenic	[23]
*P. inthanonense*	Litter of unidentified host	-	Saprobic	[24]
*P. wuyishanense*	Dead branches of unidentified host	-	Saprobic	[9]

**Table 2:** Molecular markers and their PCR primer and program used in this study.

Loci	Primer	PCR Cycle	PCR Cycle	Reference
ITS	ITS5 ITS4	GGA AGT AAA AGT CGT AAC AAG G TCC TCC GCT TAT TGA TAT GC	(95 °C: 30 s, 55 °C: 30 s, 72 °C: 1 min) × 35 cycles	[26]
LSU	LROR LR5	GTA CCC GCT GAA CTT AAG C TCC TGA GGG AAA CTT CG	(95 °C: 30 s, 52 °C: 30 s, 72 °C: 1 min) × 35 cycles	[27]
*tef1α*	EF1-728F EF-2	CAT CGA GAA GTT CGA GAA GG GGA RGT ACC AGT SAT CAT GTT	(95 °C: 30 s, 48 °C: 30 s, 72 °C: 1 min) × 35 cycles	[28]
*tub2*	Bt-2a Bt-2b	GGT AAC CAA ATC GGT GCT GCT TTC ACC CTC AGT GTA GTG ACC CTT GGC	(95 °C: 30 s, 53 °C: 30 s, 72 °C: 1 min) × 35 cycles	[29]

**Table 3:** Names, voucher numbers, and corresponding GenBank accession numbers of the sequences used in the phylogenetic analyses. Type specimens indicated with an asterisk (*).

Fungal Species	Voucher	GenBank Accession			
		ITS	LSU	*tef1α*	*tub2*
*Apoharknessia insueta*	CBS 111377 *	JQ706083	AY720814	MN271820	MG934506
*A. insueta*	CBS 114575	MN172402	MN172370	MN271821	-
*Pseudoplagiostoma alsophilae*	SAUCC WZ0451 *	OP810625	OP810631	OP828580	OP828586
*P. alsophilae*	SAUCC WZ0152	OP810626	OP810632	OP828581	OP828587
*P. bambusae*	SAUCC 1206-4 *	OP810629	OP810635	OP828584	OP828590
*P. bambusae*	SAUCC 1206-6	OP810630	OP810636	OP828585	OP828591
*P. castaneae*	SAUCCmy0162 *	MZ156982	MZ156985	MZ220321	MZ220325
*P. castaneae*	SAUCCmy0523	MZ156983	MZ156986	MZ220322	MZ220326
*P. corymbiae*	CBS 132529 *	JX069861	JX069845	-	-
*P. corymbiicola*	CBS 145052 *	MK047425	MK047476	MK047558	MK047577
*P. dipterocarpi*	TBRC 1895 *	KR994682	KR994683	-	-
*P. dipterocarpicola*	MFLUCC 21-0142 *	OM228844	OM228842	OM219629	OM219638
*P. dipterocarpicola*	MFLUCC 21-0114	OM228843	OM228841	OM219628	OM219637
*P. eucalypti*	CPC 14161	GU973510	GU973604	GU973540	GU973573
*P. eucalypti*	CBS 124807 *	GU973512	GU973606	GU973542	GU973575
*P. mangiferae*	KUMCC 18-0179 *	MK084824	MK084825	MK084822	MK084823
*P. mangiferae*	3-2.1	MN818662	MN876852	-	MW415924
*P. mangiferae*	8-1.1	MN818665	MN876855	-	MW415927
*P. mangiferae*	CDEP-48	PQ289155	PQ287499	PQ559729	PQ559735
*P. mangiferae*	CDEP-49	PQ289156	PQ287500	PQ559730	PQ559736
*P. myracrodruonis*	URM 7799 *	MG870421	MK982151	MK982557	MN019566
*P. myracrodruonis*	URM 8123	MK982150	MK982152	MK982558	MN019567
*P. machili*	SAUCC BW0233 *	OP810627	OP810633	OP828582	OP828588
*P. machili*	SAUCC BW0221	OP810628	OP810634	OP828583	OP828589
*P. oldii*	CBS 115722	GU973535	GU973610	GU973565	GU993864
*P. oldii*	CBS 124808 *	GU973534	GU973609	GU973564	GU993862
*P. perseae*	BCRC FU31382	MT233353	MT233367	-	MT251137
*P. perseae*	BCRC FU31383	MT233354	MT233368	-	MT251138
*P. perseae*	BCRC FU31384	MT233355	MT233369	-	MT251139
*P. perseae*	BCRC FU31385	MT233356	MT233370	-	MT251140
*P. perseae*	CDEP-44	PQ289157	PQ287501	PQ559731	PQ559737
*P. perseae*	CDEP-45	PQ289158	PQ287502	PQ559732	PQ559738
*P. perseae*	CDEP-46	PQ289159	PQ287503	PQ559733	PQ559739
*P. perseae*	CDEP-47	PQ289160	PQ287504	PQ559734	PQ559740
*P. variabile*	CBS 113067 *	GU973536	GU973611	GU973566	GU993863
*P. jasmini*	MFLUCC 23-0044 *	OQ786078	OQ786079	OQ850145	OQ850148
*P. inthanonense*	MFLUCC 23-0262 *	OR606510	OR633320	OR650831	OR611920
*P. wuyishanense*	HMAS 352519	PP658313	-	-	PP665724
*P. wuyishanense*	HMAS 352518	PP658312	-	-	PP665723

Abbreviations: BCRC = Bioresource Collection and Research Centre; CBS = Westerdijk Institute, Utrecht, the Netherlands; CDEP = Culture collection of Department of Entomology and Plant Pathology; CPC = Working collection of Pedro Crous housed at CBS; HMAS = Herbarium Mycologicum Academiae Sinicae, Institute of Microbiology, Chinese Academy of Sciences, Beijing, China; KUMCC = Culture Collection of Chinese Academy of Sciences, Kunming, China; MFLUCC = Mae Fah Luang University Culture Collection; SAUCC = Shandong Agricultural University Culture Collection; TBRC = Thailand Bioresource Research Center; URM = URM Culture Collection (www.ufpe.br/micoteca), Brazil.

## Data Availability

The original contributions presented in the study are included in the article, further inquiries can be directed to the corresponding author.
